# Immunopathogenesis of Different Emerging Viral Infections: Evasion, Fatal Mechanism, and Prevention

**DOI:** 10.3389/fimmu.2021.690976

**Published:** 2021-07-15

**Authors:** Betsy Yang, Kuender D. Yang

**Affiliations:** ^1^ Department of Medicine, Kaiser Permanente Oakland Medical Center, Oakland, CA, United States; ^2^ DIvision of Medical Research, Mackay Children’s Hospital, Taipei, Taiwan; ^3^ Institute of Clinical Medicine, National Yang Ming University, Taipei, Taiwan; ^4^ Department of Microbiology & Immunology, National Defense Medical Center, Taipei, Taiwan

**Keywords:** emerging viral infections, immunopathogenesis, evasion, fatality, prevention, early diagnosis, early treatment

## Abstract

Different emerging viral infections may emerge in different regions of the world and pose a global pandemic threat with high fatality. Clarification of the immunopathogenesis of different emerging viral infections can provide a plan for the crisis management and prevention of emerging infections. This perspective article describes how an emerging viral infection evolves from microbial mutation, zoonotic and/or vector-borne transmission that progresses to a fatal infection due to overt viremia, tissue-specific cytotropic damage or/and immunopathology. We classified immunopathogenesis of common emerging viral infections into 4 categories: 1) deficient immunity with disseminated viremia (e.g., Ebola); 2) pneumocytotropism with/without later hyperinflammation (e.g., COVID-19); 3) augmented immunopathology (e.g., Hanta); and 4) antibody-dependent enhancement of infection with altered immunity (e.g., Dengue). A practical guide to early blocking of viral evasion, limiting viral load and identifying the fatal mechanism of an emerging viral infection is provided to prevent and reduce the transmission, and to do rapid diagnoses followed by the early treatment of virus neutralization for reduction of morbidity and mortality of an emerging viral infection such as COVID-19.

## Introduction

Whether an RNA virus causes an endemic, epidemic, or pandemic is determined by the interactions among microorganism, host immunity, and environment. “Death or Survival” in an emerging viral infection depends largely on host immune responses because some patients are succumbed to death but most of the patients survive from the emerging infection. It is necessary to clarify varied immunopathogenesis of different emerging viral infections to prevent infection, morbidities, and mortality. Based on literature and our experiences with different emerging viral infections including enterovirus 71 encephalitis, dengue hemorrhagic fever, severe acute respiratory syndrome coronavirus-1 (SARS-CoV-1), novel influenza A(H1N1), and SARS-CoV-2 in the past 2 decades, this perspective article describes the evasion and evolution of an emerging infection among different aspects of RNA virus, environment and host, including microbial evasion and evolution (mutation, deletion, and recombination), changes of ecosystems (season, climate, and urbanization), host susceptibility, and herd immunity. “Know thyself and thy adversary to win a hundred battles”, each emerging viral infection requires individualized strategies to prevent infection and to avoid post-infectious immunopathology and fatality. A series of stepwise practical guides to infection and immunity controls are provided to prevent evasion, morbidity, and fatality of emerging viral infections.

## Evolution of an Emerging Infection on an Imbalance Between Infection and Immunity

An emerging infection is usually caused by the naive immunity of human beings encountering a novel pathogen arising from microbial mutation, vector-borne, or/and zoonotic transmission (**Table 1**) (1–12). Most of the common emerging infections are mediated by RNA viruses, which pose a higher rate of genetic mutation, sequence deletion, recombination, and reassortment of RNA virus codes (1, 13). As shown in **Table 1**, severe acute respiratory syndrome (SARS), avian flu, swine flu, and enterovirus 71 (EV71) are known to emerge from sequence mutation, deletion, recombination, and/or reassortment of RNA segments (1–6). Vector-borne diseases such as yellow fever, dengue hemorrhagic fever, and West Nile virus encephalitis are transmitted by mosquitos (**Table 1**) and affected by weather, global warming, and herd immunity (5–9). Zoonotic diseases such as Ebola, Lassa, and Hantavirus infections are affected by culture, movement of animals, and exploitation of forests (8–12).

**Table 1:** Evolution of the outbreaks of common emerging infections.

Emerging infections	Genetic changes	Vector-borne	Reservoir
**Mutation**			
Avian flu	Mutation		Birds/Ducks
Swine flu	Reassortment		Birds/Pigs
SARS-CoV-1	Deletion/recombinations		Civet cats/Bats
SARS-CoV-2	Mutation/recombinations		Pengolin/Bats
Enterovirus 71^+^	Mutation		
**Vector-borne**			
West Nile virus^+^	–	Mosquito	Birds
Dengue fever	–	Mosquito	–
Yellow fever	–	Mosquito	–
Zika fever	–	Mosquito	–
**Zoonotic**			
Ebola	–	–	Vertebrates
Lassa	–	–	Rodents
Hantavirus	–	–	Rodents

SARS-CoV, severe adult respiratory syndrome-coronavirus.

## Immune Evasion of RNA Viruses

### Mutations of RNA Viruses

Many emerging infections are caused by single-stranded RNA viruses. RNA viruses pose a higher mutation rate because its RNA-dependent polymerases usually lack a 3’-exonuclease that is present in DNA-dependent polymerases to provide proofreading ability for the genome stability during replication. RNA polymerases can produce one mutation over 10,000 replications (1, 13) and DNA viruses can produce only one mutation in 10^6^ ~ 10^8^ replications (14). The higher mutation rates of RNA viruses pose challenges to many emerging infections in human beings. For instance, influenza viruses, which are single-stranded negative RNA viruses, frequently have a nucleotide mutation causing an antigen drift responsible for periodic seasonal flu within the same host species (15). Similarly, EV71, which is a single-stranded positive RNA virus reveals varied genomic sequences in the isolates with different phenotypes (16). In an animal model, point mutations in different regions of EV71 have been attributed to different tissue tropism and fatality (17). Human severe acute respiratory syndrome coronavirus (SARS-CoV-1) caused an epidemic in Asia in 2003. SARS-CoV-1 virus mutation was estimated to be low at 0.1 per genome, similar to common single-stranded RNA viruses (18). However, SARS-CoV-2 appears to have an average of 7.23 mutations per sample (19). Single nucleotide transitions have been recognized as the major mutation of SARS-CoV-2 worldwide (19–23). The SARS-CoV-2 variant B.1.1.7 with mutation of N501Y and P681H reported from United Kingdom showed a 61% more virulence, and the variant B.1.617 with point mutations of E484Q, L452R and P681R from India revealed a 160% higher transmission rate (Ro > 5.0) (20, 21). More importantly, the variant B.1.351 with mutations of N501Y, K417N, and E484K from South Africa tended to cause breakthrough of COVID-19 vaccines showing a significant reduction in neutralization of antibodies raised by different vaccines (22, 23), potentially contributing to re-infection after natural infection or vaccination.

### Genetic Reassortment of RNA Viruses in a Cross-Species Influenza Outbreak

It is believed that the 1918 Pandemic Spanish flu that killed millions of people originated from the reassortment of cross-species virus genetic segments among avian, swine, horse, and humans (24). Flu viruses from different host animals recognize different sugar residues on respiratory and/or gastrointestinal epithelium. For instance, avian flu viruses recognize sialic acid alpha 2,3 galactose as a receptor and human flu viruses recognize sialic acid alpha 2,6 galactose as a receptor. A mutation or RNA segment reassortment of avian flu virus codes can change its hemagglutinin and recognize sialic acid alpha 2,6 galactose, thus expanding its host range to humans (25). Avian influenza uses segment reassortment of the genome to promote its cell surface binding, expand its host ranges and pose an epidemic or pandemic threat (26). These studies supported the cross-species adaptation of flu viruses through a series of reassortment events in mammals over a period of years before a pandemic outbreak (24–26), suggesting continual surveillance strategies for detection of flu viruses with cross-species genetic codes may alert to pandemics in advance.

### Nucleotide Deletion or Recombination of RNA Viruses

Coronavirus, which possesses a 3’-exonulcease to maintain a relative larger RNA genome, uses recombination and deletion to expand to and adapt in human beings (27–29). The SARS-CoV-1 likely originated in civet cats and raccoon dogs, with precursor SARS-like viruses potentially circulating in live-animal markets, and later transmitted to and adapted in humans by certain nucleotide sequence deletion (30). SARS-CoV-2 is also believed to have jumped from bats to pangolins and humans *via* a recombination of the genome in the cell binding region of spike glycoprotein (31, 32). Similarly, MERS-CoV jumped from bats to camels and humans through a series of recombinations among coronaviruses of bats, civet cats, and camels (33). Deletion of certain nucleotides in the open reading frame 8 (ORF8) has been found in SARS-CoV-2 isolates, which potentially contribute to milder infections in humans (34). Another pattern of gene deletion involved in the emerging infection of a zoonotic disease are the vaccinia-like viruses Aracatuba and Cantagalo viruses, which have been isolated from diary workers and cattle (35). The viruses have a 99% homology to the vaccinia virus but show an 18-nucleotide deletion in the A56R hemagglutinin gene (35, 36).

## Changes of Ecosystems: Season, Climate, and Urbanization

In addition to virus mutation, temperature and humidity are known to affect human-human transmission of emerging infections. Aerosol, droplet and vector-born transmissions are affected by extreme climate changes and global transportation, and zoonotic infections are affected by urbanization, moving of animals, and exploitation of forests.

### Seasonal Weather Influences Aerosol Transmission

Human seasonal influenza is usually prevalent during the winter season in which lower temperature and humidity enhance droplet and aerosol transmission. Enteroviruses are prone to outbreaks during the summer season when higher humidity enhances oral-fecal route transmission. An experimental study showed that higher temperatures and humidity block droplet and aerosol routes of influenza transmission but not close contact transmission (37). In contrast to seasonal patterns of influenza and enterovirus infection, the SARS-CoV-2 pandemic widely spread to over 180 countries in both hemispheres at the same time, suggesting that this pandemic could be related to a micro-organism that is relatively insensitive to warmer temperature and/or humidity, and can survive for a longer time on fomites, such as surfaces of handles and/or handrails. The fact that mandated face-covering, and regional or national lockdowns, even in a region of SARS-CoV-2 variant with a high reproduction number, accounts for the significantly reduced number of infections in different countries, suggests aerosol transmission as the dominant route for the SARS-CoV-2 infection (21, 38).

### Climate Changes and Global Transportation Enhance Vector-Borne Diseases

Warming temperatures and precipitation (humidity) may decrease aerosol transmission of influenza infections, but increase mosquito-borne diseases, such as Dengue fever (DF), Zika fever, Yellow fever, and Chikungunya infections which have emerged in Western and Eastern countries (39). Global transportation and urbanization may also enhance mosquito-transmitted emerging infections. These emerging RNA viruses are primarily transmitted by the mosquito *Aedes aegypti*, which originated in Africa and breeds in fresh water such as tree holes or standing water, and is now responsible for outbreaks of urban Yellow fever, dengue, and Zika fever, following the movement of larva or eggs of *Ae. aegypti* through slave trade from Africa to the New World (39–41). The relatively cold-hardy *Ae. albopictus* has moved even further north with global warming (40, 41). More than 100 countries in Africa, the Americas, the Eastern Mediterranean, South-East Asia and the Western Pacific are seriously affected by DF, with Asia representing approximately 70% of the global burden (42). Dengue fever, caused by 4 different serotypes, used to present a benign febrile illness for a century until the 1950s when a severe form of dengue called dengue hemorrhagic fever (DHF) and dengue shock syndrome (DSS) was reported in the Philippines (41). DHF/DSS spread to South America in 1981 and currently threatens countries in East Asia and South America (42). The reasons for the transition of benign DF to life-threatening DFH/DSS may be related to vector adaptation, climate change (warming and precipitation), and/or prevalence of heterotypic serotype infections (43). Like dengue, Zika virus with a mutation of NS1, transmitted by *Ae. aegypti*, has spread worldwide with a recent introduction from African and Asian lineages to the Americas (44). Zika virus causes intrauterine infection, especially in the first trimester, which can lead to congenital anomalies, particularly microcephaly, intrauterine growth restriction, and eye diseases (45).

### Urbanization and Environmental Changes Enhance Zoonotic Infections

Transition of jungle Yellow fever to endemic and epidemic urban Yellow fever is largely due to environmental changes, particularly industrialization and urbanization which enhance contacts between humans and the virus vectors in forests, as well as contacts between humans and urban virus vectors after urbanization (46). Although an effective live attenuated vaccine is available for Yellow fever, recent outbreaks in Africa and South America, where urbanization has promoted the Yellow fever virus to circulate from a jungle cycle (jungle mosquito-nonhuman primate) into an urban cycle (human-urban mosquito, *Ae. aegypti*), pose a risk to an estimated 400-500 million unvaccinated people living in at-risk areas (47). West Nile virus (WNV) is transmitted between avian hosts. The virus is transmitted by *Culex* spp. mosquitos that are infected from feeding on birds. The virus has however, expanded its geographic range from Africa, Europe, and the Mid-East to the Americas through global commerce and ecological changes (48). West Nile virus is not transmitted by a human-to-human or human-to-mosquito transmission, but rather by bird-to-mosquito-to-human transmission in which humans are the dead-end host; most of the infections are subclinical, but some can develop into severe neurological diseases, including fatal encephalitis and meningitis, particularly in older or immunocompromised patients (49). The spread of WNV north to Canada and south to Argentina indicates the growing burden of WNV in the world (50). A similar situation also occurs in Japanese encephalitis virus (JEV) transmission. JEV is an emerging flavivirus infection, transmitted by *Culex* spp. mosquitos in the Asia-Pacific region (51). JEV was initially reported in Africa and is now prevalent in the Asia-pacific region. Recently, both *Aedes* and *Culex* spp. have been shown to carry JEV in Europe (52), posing a great concern over its further spread in Northern Hemisphere countries.

Moreover, increased precipitation is associated with prevalence of Hantavirus hemorrhagic fever. Hantavirus hemorrhagic fever is transmitted by secretions of rodents and does not cause human-human transmission. The virus is found in urine and body secretion of rodents in large quantities and causes infection in humans by aerosol transmission. Hantavirus infection can lead to massive vascular damage causing “hemorrhagic fever with renal syndrome” (HFRS). HFRS was initially reported in Korea in 1950s and is now prevalent in China and Europe (53–55). The other hemorrhagic fever called “hantavirus cardio-pulmonary syndrome” (HCPS) is prevalent in the New World in North and South America (56). Recently certain overlapping hemorrhagic manifestations between HFRS and HCPS are increasingly observed (55). The HFRS has a relatively low fatality rate at about 1-3%, and the HCPS has a higher fatality rate of about 15-45%, depending on different outbreaks (53–56).

## Herd Immunity and Susceptibility of Host Variants

### Herd Immunity

Herd immunity is another key factor that determines the endemic or epidemic spread of an emerging infection. Seasonal flu is usually involved in a community where less than 10% of the population has immunity to a mutant influenza virus. Each year, human seasonal flu emerges with a certain serotype of a mutant with antigen drift resulting in an endemic or epidemic depending on herd immunity and immunization coverage. The seasonal flu, whether endemic or epidemic, usually occurs in autumn and winter when humans live in an atmosphere with a closer social distance, and lower temperature and humidity. The flu epidemic can be limited by herd immunity and/or mass vaccination that is selected and prepared from the emergence of seasonal influenza in the previous years. This is an example of the balance between virus mutation and herd immunity (57). A seasonal flu usually has a reproduction number (Ro) about 1.2~1.3, which can be controlled by herd immunity or vaccination if coverage is over 25% of the population (1 - 1/Ro = 1 - 1/1.3 = 25%). A flu pandemic is different and is usually caused by a series of antigenic reassortments (shifts) among cross-species flu viruses, which is novel to a population without immunity and causes a potential pandemic and fatal transmission. A novel cross-species flu virus usually causes a pandemic involving about 30-50% of the population in the initial years because almost all humans are susceptible to the novel influenza virus (58). This pandemic could re-emerge after a period of several years or decades; approximately 36 years (58), depending on the evolution and adaptation of a cross-species flu virus among avian, swine, and human hosts, and on the control of school closures, vaccination, facemask use, and isolation (59, 60). Another hypothesis for pandemic re-emergence is related to introduction of a dominant flu subtype virus into a population where the kinetic balance between virus virulence and human immunity is broken. Once a novel strain of flu virus can cause human-human transmission, it usually has a Ro value around 1.8 in the first wave of the epidemic and an attack rate of 10-30%. Second and/or 3^rd^ waves will follow until herd immunity of over 60% is reached (60). The novel strain virus eventually transforms into a dominant subtype of the influenza epidemic and affects most of the population, particularly children who become infected with the pandemic strain over several years. This will confer some level of protection to older individuals and protect them from morbidity and mortality of influenza until the next pandemic (60). In a simulation model, the Ro of a novel influenza virus transmission among human-human transmission is around 1.3~1.8 (61), and that of the SARS-CoV-2 is around 2.3 (62), respectively. To control the pandemic requires infection or immunization rates of 33% (1 – 1/1.5) and 57% (1 – 1/2.3), respectively, based on the equation, 1 - 1/Ro, to cease the pandemic (63). Although a number of SARS-CoV-2 vaccines have been shown effectiveness on controlling the outbreaks with different SARS-CoV-2 variants, certain variants cause higher virulence, higher reproduction number, and/or breakthrough of COVID-19 vaccines (20–23), potentially contributing to re-infection after natural infection or vaccination. Whether the novel SARS-CoV-2 pandemic might also cause periodic waves of epidemics remains a great concern (64).

### Genetic Polymorphisms Associated With Infectivity and Immunopathology

Genetic polymorphisms of immunity genes and virus receptors also affect infectivity and fatality of an emerging infection. Polymorphisms of CCR5, CCR2, CX3CR1, and SDF1 have been shown to influence HIV susceptibility and treatment responses (65). Polymorphisms of human leukocyte antigen (HLA), MBL2, CD209, and vitamin D receptor genes were associated with development of TB in HIV patients (66). We have found that a combination of TGFβ and CTLA-4 genotypes was significantly associated with the susceptibility to DHF (67). We (68) and others (69) have shown that a promoter polymorphism of CD209, a C-type lectin, was significantly associated with DHF. Recently, we found that the L-SIGN (CD299) polymorphism at the neck region of 9-tandem repeats was associated with susceptibility to DHF and correlated to virus replication and immune response (unpublished data). Similarly, the nine-repeat of CD299 isoform was associated with increased HIV viral load and HIV sexual transmission (70). ACE2 is a receptor for SARS-CoV-1 and SARS-CoV-2 infection, but the polymorphism of ACE2 was not associated with severity of infection (71). In contrast, glycosylation of Spike antigen is critically involved in recognition and binding of coronavirus (72) and affects binding affinity of host antibodies (73). TLR7 genetic variants cause predisposition to severe COVID-19 infections (74). Genetic variants in IL6R, TLR3, and DC-SIGN genes were associated with susceptibility and/or severity of DF (75). Genetic polymorphisms of DC-SIGN, TLR3 and TNF-α genes are also risk factors for the susceptibility and disease progression of Chikungunya infection (76). Interferon-inducible transmembrane protein 3 (IFITM3) gene is associated with susceptibility to severe influenza (77), and the variant with higher TMPRSS2 expression confers a higher risk to susceptibility of human A(H7N9) influenza and severity of A(H1N1)09 influenza (78). Ran Binding Protein 2 (RANBP2) gene mutations increase the susceptibility to recurrent episodes of necrotizing encephalitis with respiratory viral infections, particularly influenza infection (79).

### Culture, Occupation, and Social Events

Culture, occupation, and socioeconomic status also affect the spread of emerging infections. A patient with hemorrhagic fever and symptoms of bloody diarrhea, bleeding gums and skin, hemorrhagic eyes or urine, should be traced back to the suspected contact of Ebola virus or Marburg virus through a dead or sick animal (for Ebola virus) or a mine or cave with bat colonies (for Marburg virus) (80). Ebola and Marburg hemorrhagic fevers usually begin as an exposure to affected animals followed by human-human transmission (80). Travelers who visit the endemic area of Africa may spread the filoviruses worldwide (81). Health caregivers or people in diagnostic laboratories who come into contact with tissue fluid samples may become infected through human-human transmission of Ebola virus because the virus shedding time in tissue fluids can persist for 30 to 60 days (82). Ebola outbreaks are also related to cultural funeral ceremonies, including washing and touching the corpse and close contact during funeral ceremonies (83). Another cultural issue that influences emerging infections is wet markets in Asia. SARS coronaviruses and avian influenza viruses can be identified in live poultry markets (84), posing a need for virological and serological monitoring of viruses and hosts in live poultry markets which are still popular in Asian countries.

## Classification of Immunopathogenesis of Different Emerging Infections

An emerging infection can rapidly lead to a pandemic with high fatality rates. Each individual emerging infection has its unique pattern of infectivity related to virus-host interactions underlying ligation of pathogen-associated molecular pattern (PAMP) to pattern recognition receptor (PRR) for the signaling of immune responses toward proper defense or morbidity. It is always debatable whether the high fatality of an emerging infection is related to viral virulence, immune deficiency, or immunopathology.

**Figure 1:**
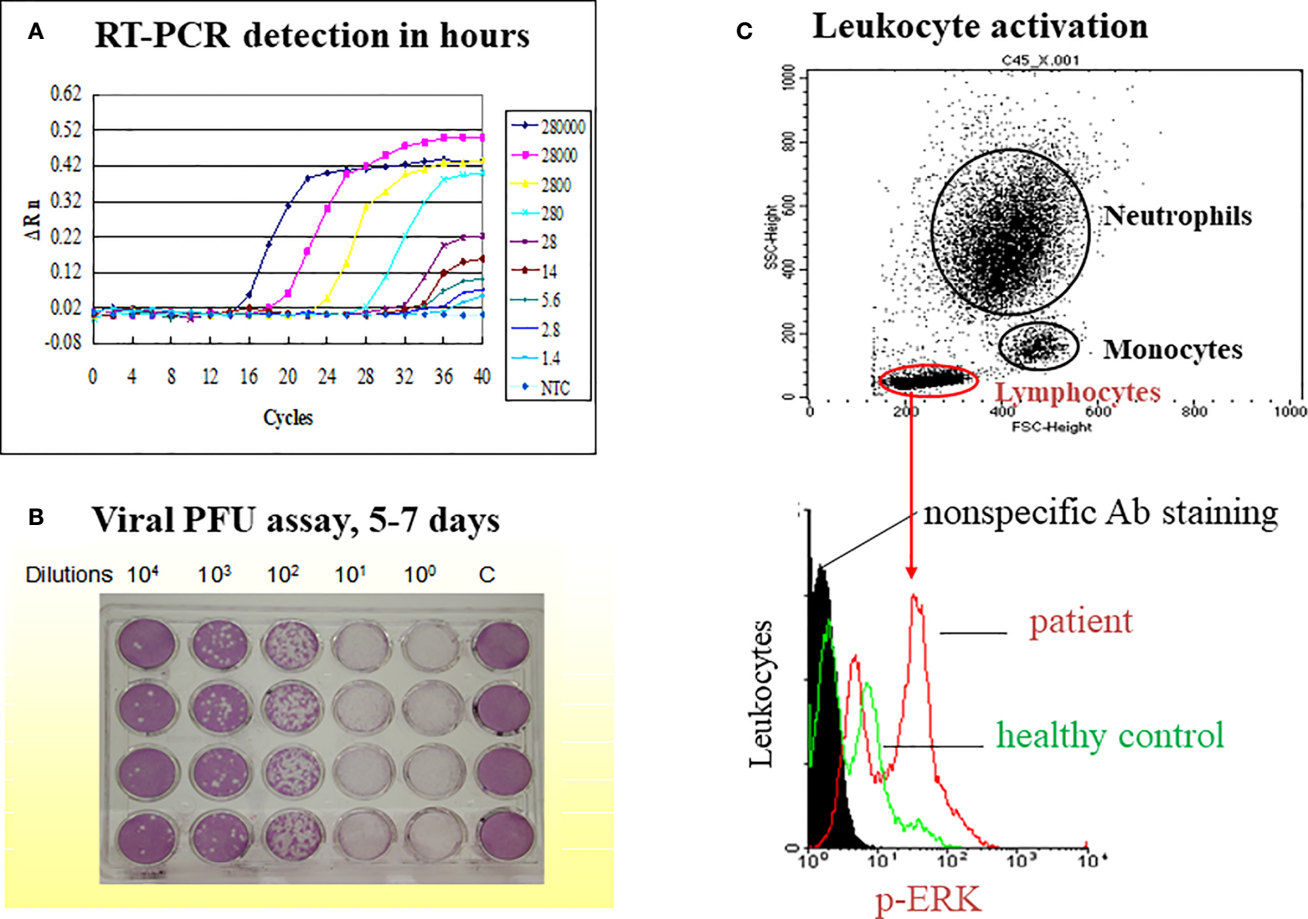
A model on simultaneous measurement of viral load and immune responses in an emerging infectious disease such as dengue fever. The study model presented is derived from our previous publication (Yeh, et al. *FEMS Immunol Med Microbiol.* 2006;48 (1):84-90). **(A)** A TaqMan qRT-PCR is used to replace classical time-consuming plaque-forming unit assay of viral load **(B)**. The limit of detection (LOD) is 14 copies of the dengue 2 virus while cutting off the PCR cycle at 35, and the LOD is 1.4 copies of the dengue virus while cutting off the PCR cycle at 40. **(C)** A flow cytometric assay is used to gate different populations of leukocytes (neutrophil, monocyte and lymphocyte) for the activation assay such as ERK activation in lymphocytes as indicated.

We studied immune responses to enterovirus 71 (EV71) (85–88), dengue (67, 68, 89–96), SARS-CoV-1 (30, 97–100), and influenza A (H1N1) 2009 infections (101–104) employing a real time simultaneous detection of viral load and immune responses (**Figure 1**). A TaqMan qRT-PCR was used to replace classical time-consuming plaque-forming unit assay of viral load, and cell cytometers were used to measure quantity and quality of leukocyte counts and activation. Based upon our studies and others’ studies, we have classified common immunopathogeneses of different emerging infections into 4 categories in **Table 2**: 1) Deficient immunity with disseminated viremia; 2) Pneumocytotropism with/without later hyperinflammation; 3) Augmented immunopathology; and 4) Antibody-dependent enhancement of infection with altered immunity.

**Table 2:** Mechanisms of different emerging infections.

Diseases	Immunity	Tissue response	
		Regional	Systemic
Mechanism 1: Defective immunity with systemic dissemination			
Ebola	B cell defect	Hemorrhage	Shock
Lassa	T cell defect	Hemorrhage	Shock
Enterovirus 71	T cell defect	Neurotropism	Brain-pulmonary Syndrome
WNV	B cell defect	Neurotropism	Encephalitis
Mechanism 2: Pneumocytotropism with/without hyperinflammation			
SARS-CoV-1	Proinflammation	Pneumocytotropism	ARDS
Swine flu	Immunosuppression	Pneumocytotropism	ARDS
SARS-CoV-2	Proinflammation	Pneumocytotropism	Hyperinflammation
Mechanism 3: Augmented immunopathology			
Hantavirus	Augmented inflammation	Renal/lung damage	Shock/ARDS
Avian Flu	Augmented inflammation	ARDS	Hemophagocytosis
Mechanism 4: Immune cross-enhancement of infection with altered immunity			
Dengue	Antibody-dependent	Hemorrhage	Shock
Ross River virus	Antibody-dependent	Rashes	Polyarthritis

Abbreviations used: WNV, West Nile virus; ARDS, acute respiratory distress syndrome; SARS, severe acute respiratory syndrome.

### Deficient Immunity With Disseminated Viremia

Emerging infections that fit into this category include Ebola, Lassa fever, West Nile virus (WNV) encephalitis, and EV71 encephalitis (**Table 2**). A study with Ebola and Lassa viruses showed that Ebola and Lassa virus infection could compromise monocyte-derived dendritic cell function resulting in impaired adaptive immunity (105). Patients with fatal Ebola infection tended to have an impaired humoral response associated with 100% detectable viremia (106, 107). Lassa fever with fatal outcome was related to impaired T cell reaction associated with overt viremia and disseminated vascular insults (108, 109). For WNV encephalitis, the virus tended to infect immunocompromised hosts, especially those with B cell defect, causing higher mortality (110).

**Figure 2:**
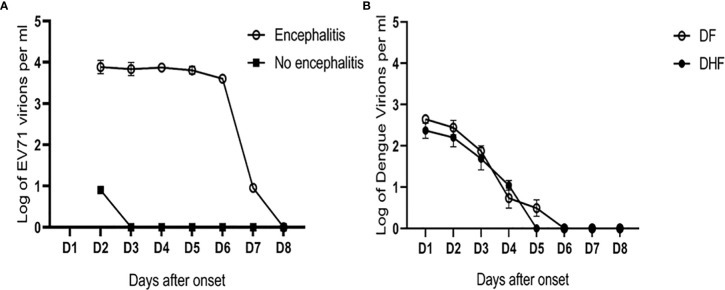
Different patterns of viral load in EV71 and dengue infections. **(A)** The blood viral loads in EV71 encephalitis patients were significantly higher than those with no encephalitis (data derived from 12 pairs of case-control samples). Based on the unit of one milliliter blood (ml), the limit of detection (LOD) is 9 copies/ml in patients with EV71. In contrast, **(B)** the blood viral loads in patients with dengue hemorrhagic fever (DHF) were not significantly different from those in dengue fever (DF). The LOD is 3 copies/ml in patients with dengue infection (the representative graph is derived from the publication (Chen, et al. *FEMS Immunol Med Microbiol.* 2005;44 (1):43-50).

Our study on the immunopathogenesis of EV71 encephalitis also demonstrated that younger children with impaired T cell activation of CD40L were associated with EV71 infection complicated by encephalitis (85). Patients with EV71 encephalitis tended to have higher IL-8 and IL-2 levels than those without (86). Patients with encephalitis associated with neurogenic pulmonary syndrome had augmented IL-6 and TNFα levels in their blood (87). Further studies showed sialylated glycans as a receptor and inhibitor of EV71 infection to DLD-1 intestinal cells (88). The blood viral load in EV71 encephalitis patients was significantly higher than in those without encephalitis (**Figure 2A**). In contrast, the blood viral load in patients with dengue hemorrhagic fever (DHF) was not significantly different from patients with dengue fever (**Figure 2B**). Taken together, the severity of Ebola, Lassa fever, West Nile encephalitis and EV71 encephalitis is correlated to immune deficiency with disseminated viremia. Detection of definite impaired immunity and/or viremia in these infections alerts to the seriousness and calls for emergent medical assistance.

### Pneumocytotropism With/Without Later Hyperinflammation

Emerging infections fit into this category include SARS-CoV-1, SARS-CoV-2, and swine influenza A(H1N1)2009 which bind and fuse into the cells of respiratory tract and cause proinflammatory reaction in the lungs, called pneumocytotropism (**Table 2**). SARS-CoV-1 and SARS-CoV-2 are believed to infect the human respiratory tract by binding to angiotensin-converting enzyme 2 (ACE2) (111), and influenza A virus recognizes sialic acid alpha 2,6 galactose on respiratory epithelial cells as a receptor (23). The viruses enter the lung epithelial cells and induce innate immunity with production of interferons which limit viral replication before adaptive immunity. In different virus-host interactions, the virulent antigen(s) of the viruses (112), or host genetic variants (74–78), could impair the innate immune response and cause proinflammation or immunosuppression, followed by altered hyperinflammation with skewed Th17 reaction (113, 114). The viral RNA of SARS-CoV-1 and SARS-CoV-2 cannot only be detected in respiratory secretions but also in urine, feces, tears, and blood (115, 116). Virus shedding is not apparent during the incubation period in SARS-CoV-1 but can persist for 15-20 days after illness onset (115). However, virus shedding of SARS-CoV-2 virus is found in nasopharyngeal swabs before symptom onset and can persist for at least 3 weeks (116). The RNA virus was found in blood and urine of SARS-CoV-2 patients, but the urine or blood samples never yielded the virus from culture (116). In SARS-CoV-2 infections, the predominant pattern of lung lesions in autopsy is ARDS, similar to the findings in other two coronavirus, SARS-CoV-1 and MERS-CoV, showing capillary congestion, hyaline membrane, interstitial edema, pneumocyte hyperplasia and platelet-fibrin thrombi, associated with infiltration of macrophages in alveolar lumens, and lymphocytes (117, 118). Electron microscopy revealed viral particles in cytoplasmic vacuoles of pneumocytes. Pathogenesis of the deaths in cardiopulmonary events of COVID-19 patients are not due to pneumonia with ARDS at all, but some sort of thrombosis or disseminated intravascular coagulopathy (DIC) which occurred before death (119). Patients with severe COVID-19 have a hyperinflammation with higher plasma IL-2, IL-7, IL-10, G-SCF, IP-10, MCP-1, MIP1A, and TNFα levels, particularly in elders showing “inflamed-aging” (120). Elders with SARS-CoV-1 or SARS-CoV-2 infections had a higher mortality in an age-dependent correlation, and in an association with co-morbidities (121–123).

**Figure 3:**
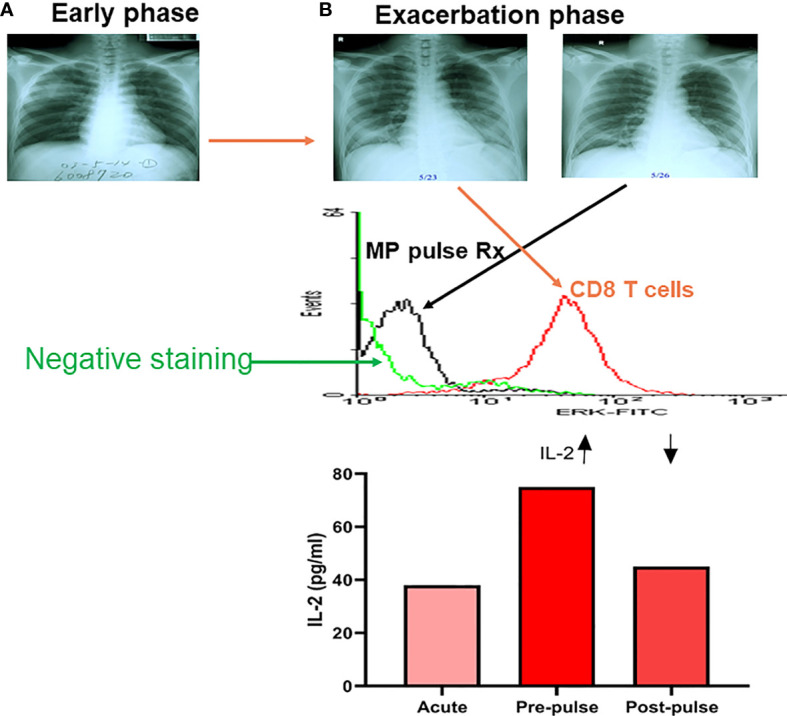
A kinetic tracing of chest X-rays, intracellular p-ERK and plasma IL-2 levels in a patient with late exacerbation of SARS-CoV-1 before and after methylprednisolone pulse treatment (MP pulse Rx). An early phase X-rays film in a SARS patient **(A)**, who developed late phase exacerbation **(B)** showing a high intracellular p-ERK level of CD8 T cells (flow cytometric analysis of intracellular phosphorylated ERK levels) in exacerbation, and dramatically decreased after a 3-day course of methylprednisolone (MP; 1 gm/day) pulse Rx, associated with a decrease in plasma IL-2 level after the MP pulse Rx. (Data presented are derived from Li & Yang, et al. *J Immunol.* 2004;172 (12):7841-7).

We found that one-third of SARS patients had detectable blood SARS-CoV-1 RNA, although the viremia was unlikely related to the outcome of the disease (97). Patients with SARS-CoV-1 infection had a significant higher IL-8 level associated with augmented phosphorylated p38 expression of CD14 cells and depressed phosphorylated p38 expression of CD8 T cells in early stage (<7 days) but higher IL-2 levels in late stage (>7 days) (97, 98). One of the 15 SARS patients studied had a late exacerbation of ARDS with a surge of p-ERK expression of CD8 T cells requiring steroid pulse therapy, which reversed the hyperactivation of p-ERK expression after the steroid pulse therapy (**Figure 3**). An exposure history and an early progression of chest X-rays in SARS-CoV-1 patients was associated with poor outcomes (99). SARS patients tended to have lymphopenia and thrombocytopenia which was caused by cell apoptosis associated with higher sFasL levels, and vascular sequestration associated with increased sVCAM-1 levels (100). Taken together, these results suggest uncontrolled regional pneumocytotropic lung damage, but not viremia responsible for the poor outcome of SARS-CoV-1. Some SARS-CoV-1 patients revealed a secondary exacerbation between the second and third weeks of infection in which CD8 T cell activation with higher IL-2 production was found (**Figure 3**).

**Figure 4:**
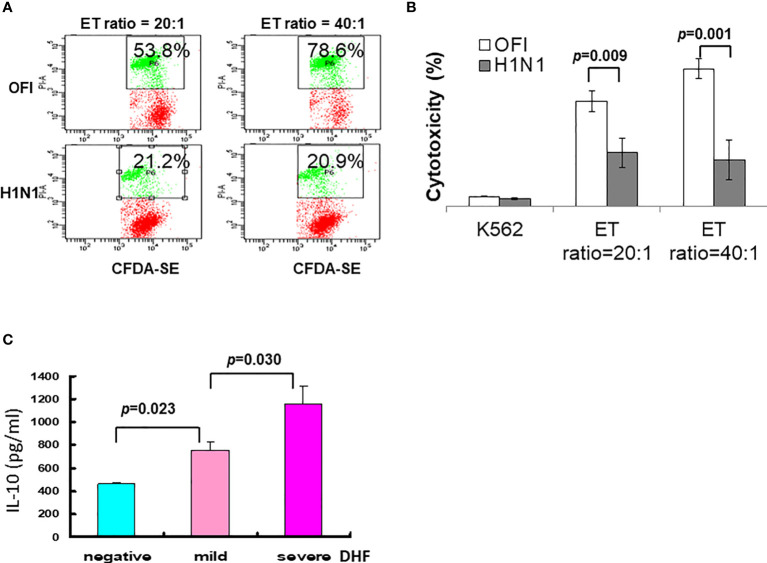
Immune alteration of influenza A (H1N1) 2009 and dengue infection. **(A)** Depressed NK cell activity in influenza A (H1N1) 2009 infection. Employing K562 cells labeled with CFDA dye, the cell cytotoxicity was detected by propidium iodide (PI). **(B)** The NK cell cytotoxic activities in patients with influenza A (H1N1) 2009 infection were significantly depressed either in effector-target (ET) ratio at 20:1 or 40:1 in comparison to those in patients with other febrile illness (OFI). **(C)** A significant association of IL-10 levels among dengue infections with mild or severe DHF. In a dengue outbreak, a cohort of patients with dengue fever, and dengue hemorrhagic fever mild or severe showed a significant higher level of IL-10 toward severe DHF (data derived from Chen RF, Yang KD, et al. *Trans R Soc Trop Med Hyg.* 2007;101 (11):1106-13).

In the swine influenza A (H1N1) 2009 outbreak, we found different clinical features between children and adults (101–103), and the younger children had a longer viral shedding time (102), and characteristic early lymphopenia and lower C-reactive protein levels (103). The influenza A (H1N1)2009 infection was associated with depressed NK cell function (**Figures 4A, B**). In other words, a higher initial viral uptake and/or suppressed immunity determines whether there is overwhelming regional lung damage and complication or not.

### Augmented Immunopathology

Certain viruses do not cause systemic virus dissemination in the blood, but cause a systemic immune response with cytokine storm, or indirectly assault vessels by augmented immune reactions resulting in hemorrhage or vascular leakage (124–126). Emerging infections fit into this category, including Avian flu and Hanta viruses (**Table 2**). Patients with fatal H5N1 infections had a cytokine storm with low peripheral blood T-lymphocyte counts, associated with pharyngeal viral loads (127). Patients with fatal Hantavirus fever renal syndrome (HFRS) or Hanta cardiopulmonary syndrome (HCPS) had varied cytokine storms without viremia (128). Currently, H5N1 avian flu virus infects humans *via* the bird-to-human transmission and likely by the oral-fecal route, but not *via* aerosol transmission (129). However, avian H5H1 flu virus RNA was detected by RT-PCR in the lungs, intestines, and spleen. Active viral replication was limited to the lungs and intestine. This is compatible with clinical symptoms of pneumonia and diarrhea associated with altered immunity with circulating thrombocytopenia, cytokine storm and hemophagocytic syndrome (130). This suggests that regional unlimited viral replication due to depressed immunity, which is associated with uncontrolled proinflammatory cytokine production, is involved in the immunopathogenesis. An appropriate treatment may require not only an anti-viral agent (e.g. Tamiflu for avian flu within 3 days), but also immunomodulation of cytokine storm (e.g. anti-IL6 for COVID-19) as early as possible.

Hantavirus infects humans exposed to secretions of reservoir hosts (e.g., rats), resulting in a dead-end infection in humans with a long incubation period between 2-4 weeks. The hantavirus replicates in endothelial cells without cytopathic effect (CPE) but induces vascular leakage by a mechanism related to anti-viral mediators of endothelial cells, or cell immunity directed against infected cells by different cytokine storms in blood and affected tissues (56, 131). Infections occurring in the lungs are called HCPS (55, 56), and those occurring in the kidneys are called HFRS (53, 54). Hantaviruses infect endothelial cells *via* the β3 integrins which induce hyperresponsive to the permeability of endothelial cells by VEGF (131).

### Antibody-Dependent Enhancement of Infection With Altered Immunity

The human immune system can discriminate non-self-microbes and raise a memory immune reaction after the infection. The memory immune response produces neutralizing antibodies for immuno-surveillance of the same microbes and/or cross-reactive protection of similar microbial infections. Unfortunately, certain emerging infections that raises antibodies may cause cross-enhancement of infections as seen in dengue fever and Ross River viral infections (**Table 2**). Patients with secondary dengue fever are more susceptible to complications of DHF and dengue shock syndrome (DSS) (132–134). The antibodies raised in primary dengue infection can circulate in the blood for years or even decades, providing protection from the same serotype of dengue infection, but cause cross-enhancement of secondary heterotypic dengue infections, in which subneutralizing antibodies enhance heterotypic dengue virus infection (89–92), and alter immune response shifting type 1 T helper (Th1) response to Th2 response with dominant IL-10 in patients with DHF (89, 92, 94). The first implication for DHF was the observation that over 85% of children with DHF had high dengue heterotypic cross-reactive antibody titers in a Bangkok outbreak of DHF (43, 132), suggesting an antibody-dependent enhancement (ADE) of dengue infection in the pathogenesis. This hinders dengue vaccine development because of antibody-dependent enhancement (ADE) of dengue infections due to vaccine-induced heterotypic antibodies. In contrast to the DHF, which more frequently occurs to children in East Asia, our studies found that elders with comorbidities are more susceptible to DHF (90, 91, 95), and patients carrying certain genotypes were significantly associated with DHF (67, 75). We also found that previous subclinical dengue infections are more frequently associated with DHF (92, 94, 96), and elders with comorbidity or concurrent bacteremia have a higher mortality (91, 95). To explore the biomarker for early detection of DHF, we found that blood IL-10 levels were significantly associated with severity of DHF (**Figure 4C**). In addition to the ADE of dengue infections (43, 89, 92), another example of ADE was demonstrated in Ross River viral infections with polyarthritis, in which the presence of antibody enhances viral infection by macrophages (135). Like ADE of dengue, antibodies of COVID-19 infections have been proposed to induce augmented immune response by Fcγ-receptor mediated enhancement (136).

## Strategies to Prevent Fatality Based on Mechanistic Signatures of Immunopathogenesis

Mechanisms of the fatality in various emerging infections are different so that protection from fatality of each emerging infection requires an advanced deployment on early detection of the fatal pathogenesis among viral dissemination, immune deficiency, and immunopathology to develop a proper strategy to prevent or decrease fatality. For those with disseminated viremia, anti-viral agents such as interferons, inhibition of RNA replication with drugs such as remdesivir or favipiravir, and/or agents that block viral shedding, such as silmitasertib can be applied (137–139). Those with immune deficiency or with high viral load require earlier supplementation of hyperimmune immunoglobulins, neutralizing MoAbs, or convalescent plasma from convalescent patients (140–142). Those with immunopathology such as cytokine storm require administration of cytokine antagonist, inhibition of complement cascade, or adsorption of circulating cytokines (143–145). Those with infection-associated hemophagocytosis, also called secondary hemophagocytic lymphohistiocytosis or macrophage activation syndrome, require administration of IVIG, cyclosporin-A, corticosteroids, and/or anti-cytokine therapy (146, 147). As shown in **Table 3**, we have summarized how to differentiate fatal mechanisms and early signature markers for crisis management of early recognition and prevention, based on pathogenic mechanisms of overt viremia, tissue-specific organ failure, cytokine storm, and iatrogenic insults.

**Table 3:** Early recognition of fatal mechanism for prevention of fatality.

Fatal Mechanisms	Early Recognition	Prevention of fatality
**1. Systemic viremia**	**Early detection of viremia**	**Early anti-virus & ring vaccination**
Lassa fever	RDT ≧ qPCR > ELISA	Rivavirin or convalescent plasma
EV71	qPCR of blood & saliva	IVIG
Ebola	qPCR of blood & stool	REGN-EB3, MoAbs, & Ring vaccination, rVSV-ZEBOV
**2. Cytotropic organ failure**	**Host response & genotype**	**Early organ protection**
HCPS	PaO2/FiO2, neutrophilia	ECMO/CRRT, steroids
HFRS	Creatinine, cytokines	CRRT/Icatibant
Necrotizing encephalitis	Host RANBP2 and IFITM3 mutations	Prophylactic oseltamivir
(Fulminant influenza)		
**3. Cytokine storm**	**Immunopathology assays**	**Immunotherapies**
Hyperinflammation	IL6, IL8, TNFα, IL1β	Anti-IL-6, anti-IL1, CRRT
Shock, Coagulopathy	D-dimer, low platelets	ECMO, LMWH, anti-C5a
Hemophagocytosis	Ferritin, sCD25, anemia	IVIG/steroids, Cyclosporin
**4. Superposition**	**Microbial/metabolic factors**	**Integrated therapies**
Sepsis	MS fingerprinting	Anti-virus & anti-bacteria
Nosocomial infections	Comorbidities	Containment, protection, RDTs, MoAbs, and plasma therapy
Iatrogenic side-effects	Drug toxicity/interactions, pipeline clogging, overload of health providers, shortage of medical supplies	Monitor of drug levels, continuing education, practicing virtual reality, advance deployment

RDT, rapid diagnostic test; qPCR, quantitative polymerase chain reaction; ELISA, enzyme-linked immunoassay; ECMO, extracorporeal membrane oxygenation; MoAbs, monoclonal antibodies; CRRT, continuous renal replacement therapy; sCD25, soluble CD25; LMWH, low molecular weight heparin; RANBP2, Ran Binding Protein 2; IFITM3, interferon-inducible transmembrane protein; IVIG, intravenous immunoglobulin; MS, mass spectrometry.

### Early Detection of Viremia for Reducing Viral Spread and Fatality

An emerging infection that causes impaired or delayed cell immunity or production of neutralizing antibodies can raise systemic viremia or immunopathology that causes a high fatality with hemorrhagic fever (coagulopathy), respiratory failure, and/or encephalitis. Patients with fatal Ebola infection tend to have 100% detectable viremia (106, 107). The early recognition of infection, viremia or antigenemia could promote not only an early administration of neutralizing antibodies (MoAbs or convalescent plasma) for reducing viral load and fatality, but also a timely interruption of the transmission of the emerging infection (**Table 3.1**). For instance, a rapid diagnostic test (RDT) for the Lassa viral antigen by a point of care test of immunochromatography can alert for systemic viremia (148). Detection of an early systemic viral load of Lassa fever in blood will raise the warning sign for early intervention (149) with administration of ribavirin (anti-viral agent), and/or convalescent plasma. These early interventions were shown to significantly reduced fatality (150, 151). Our study on the immunopathogenesis of enterovirus 71 encephalitis also demonstrates that younger children with impaired T cell reaction are associated with delayed CD40L expression and viremia (85, 86). In a simulation for Ebola containment based on a Ro value of 2.0, it is estimated that a rapid blood test reduces the attack rate from 80% to nearly zero, and the average diagnostic time from 5 days to 1 day in 60% of Ebola virus-infected patients (152). More importantly, in an Ebola outbreak, the early diagnosis would also promote the efficacy of ring vaccination by rVSV-ZEBOV which provided 100% vaccine efficacy (0/4539 *vs.* 39/4557 cases) in the immediate vaccinees after known exposure compared to the delayed group vaccinated 21 days after exposure (153). Administration of MoAbs or convalescent plasma in early stage of infection has also been shown effectiveness on the limitation of disease progression in Ebola (140), SARS-CoV-1 (141), MERS-CoV (142), and SARS-CoV-2 (154).

### Rapid Diagnosis for Preventing Cytotropic Organ Failure

Rapid diagnoses of emerging viral infections using point of care tests (POCT) for detection of specific antigen or nucleotide are made available in recent years, particularly during the COVID-19 pandemic (155). The paper-based POCT can be done in 15 minutes by detecting antigen-antibody reaction in secretion of upper respiratory tract or blood (155). The early detection within 3 days may be followed by early treatment of neutralizing antibodies to reduce viral load of the lung and reduce complication (140, 154). Some emerging infections can cause tissue-specific cytotropism; for instance, SARS-CoV-2 and Avian influenza virus can cause respiratory distress syndrome and Hanta virus can cause renal failure. The emergence of Avian flu and Hantavirus syndrome did not cause systemic viral dissemination, but assaulted vascular endothelium by augmented immune reactions, resulting in hemorrhage, pulmonary edema, or renal failure (125, 126). As shown in **Table 3.3**, kinetic monitoring of lung and kidney functions is mandatory to prevent Hantavirus-induced organ failure. This can be accomplished through ventilation support, continuous renal replacement therapy (CRRT), and/or extracorporeal membrane oxygenation (ECMO) support (156). In addition, Icatibant which blocks the binding of bradykinin has been used to treat hantavirus infection with complement activation and coagulopathy (157). For patients with a fulminant or a treatment resistant course, strategies to identify host genetic variants that compromise defense, or to identify viral virulent factors that induce immunosuppression are required. For instance, a respiratory tract infection with repeated influenza infections or fulminant (necrotizing) encephalitis should be screened for genetic mutations at Ran Binding Protein 2 (RANBP2) (79) or interferon-inducible transmembrane protein 3 (IFITM3) (77), respectively, and anti-virus treatment (e.g. Tamiflu) should be initiated as early as possible.

### Targeting Cytokine Storm by Immunotherapies

Certain emerging infections can cause altered immunity which results in the release of untoward cytokines causing cytokine storm of immunopathology. Because organ failure is related to inflammatory insults, anti-inflammatory regimens are necessary (**Table 3.3**). The cytokine storms in different emerging infections are frequently associated with augmented levels of IL-6, IL-1β, IL-8, TNFα, and/or IP-10 (87, 89, 92, 97, 120, 158). Anti-IL6R and/or anti-IL1 antibodies are indicated in the treatment of cytokine storm of COVID-19 (159). Moreover, the cytokine profiles induced by coronavirus infections are related to T helper cell type 17 (Th17) reactions (97, 114, 120, 158, 159), to which immunoregulatory therapies have been proposed (113, 160). For cases complicated by abnormal complement cascade and coagulopathy (higher D-dimer and lower platelets), a combined therapy with anti-C5a antibody and Jak1 inhibitor may be needed (161). In addition, some patients may require utilization of heparin, ECMO and/or CRRT treatment (162). Avian flu with cytokine storm might be associated with augmented immune responses such as hemophagocytosis showing anemia, thrombocytopenia, hyperferritinemia, hypertriglyceridemia, and adult type respiratory distress syndrome (ARDS) without detectable viremia, which may require a combination of IVIG with steroids, and cyclosporin A or etoposide (163, 164).

### Prevention of Superimposed and Iatrogenic Morbidity

An emerging infection can cause high fatality when conditions such as sepsis and complications due to comorbidities or malpractices are superimposed (**Table 3.4**). Strategies to alert to these superimposed conditions will promote integrated therapies including anti-virus, anti-bacteria, anti-inflammation, and ventilation or renal support. Many patients with an emerging infection die of sepsis because of virus-induced immunosuppression (95, 165). In these cases mass spectrometry fingerprinting of blood culture is necessary to early detect bacteremia, identify antibiotic resistance, and prevent sepsis. New drugs or crisis management may result in novel toxicity or unexpected drug interactions in patients with comorbidities. Certain emerging infections, particularly those prone to nosocomial infections such as Ebola and SARS, can impact not only the general population but also health care providers and medical institutions. Containment of nosocomial and emerging infections in health care centers and long-term care facilities where elders are frequently bedridden with multiple comorbidities is especially important, since co-morbid patients are usually super-spreaders and succumb to higher morbidity and mortality, requiring early RDTs and reduction in viral load by MoAbs or convalescent plasma. Overtreatment or undertreatment of an emerging infection may cause iatrogenic morbidity and mortality. For instance, early mechanical ventilation or late use of neutralizing antibodies may increase morbidity and mortality. Shortage of medical resources or shortages of health providers could also increase potential complications. Continuing education with advanced deployment and use of computer simulation can be used to reduce iatrogenic side effects.

## Summary

**Figure 5:**
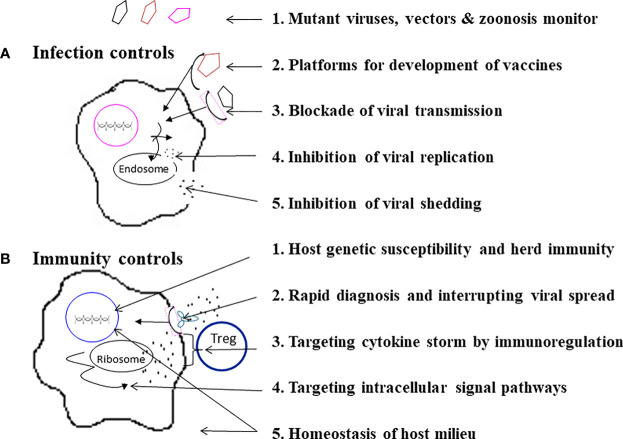
A stepwise practical guide to do infection controls and immunity controls. As specified, **(A)** Infection controls can be made by a 5-step program, and **(B)** Immunity controls can be approached by another 5-step program.

Because each individual emerging infection has its own evolutionary trait, transmission route, and immunopathogenesis, each emerging infection requires individualized strategies to prevent infection, morbidity and mortality. However, “stones from other hills may serve to polish the jade of this one,” advance deployment may be made for mitigating a pandemic and reducing fatality. A stepwise guideline for infection and immunity controls to prevent an emerging infection may be possible (166). As shown in **Figure 5**, there are 5 check points of infection controls to prevent infection, morbidity and fatality:

(1) Monitoring for mutant viruses, vectors & zoonosis. The best way to prevent pandemics and fatalities due to an emerging infection is to monitor potential emerging microbials in mutations, vectors, and zoonosis before and during pandemics (1–13, 27–30, 84). In this era of a global village and changes of ecosystems, early prediction, recognition, and elimination of an emerging infection is not guaranteed. Preparedness of mass vaccination, convalescent plasma and specific anti-virus agents is also important.

(2) Platforms for development of vaccines. A couple of new platforms for rapid development of vaccines by avirulent virus vectors with DNA, mRNA vaccine and recombinant protein technologies that are safe and efficacious have been made possible (167–170). For instance, the fast pipelines of vaccines for an emerging infection such as COVID-19 were made available within one year (169, 170).

(3) Blockade of viral transmission. Before a vaccine is available for an emerging infection, it is important to encourage wearing of facemasks, keeping social distance and doing surface disinfection. These measures may not only have an effect on blocking transmission of the emerging infection, but may also have collateral benefits by decreasing other upper respiratory tract infections (171, 172).

(4) Inhibition of viral replication. The inhibition of viral replication could be made by antiviral agents directed against virus-cell fusion, virus and host proteases, and RNA synthetase (137–139).

(5) Inhibition of viral shedding. In SARS-CoV-2 infections activations of casein kinases (CK2) and protein kinases (MAPK) have been demonstrated (137). Inhibitors of CK2 and protein kinases which have demonstrated safety data in human trials have been proposed to re-purposing of the FDA-approved kinases inhibitors for the treatment of COVID-19 (137, 173, 174). A combination of anti-viral replication and shedding may provide a synergistic effect on mitigation of viral transmission

There are 5 other check points for immunity controls of an emerging infection:

(1) Host genetic susceptibility and herd immunity. In different emerging infections mortality ranges from 1% to 60%. Many humans survive because of host immunity and herd immunity. For patients who experience a fulminant disease course or treatment resistance, it is necessary to survey for host genetic susceptibility. For instances, deletion or mutation of TLR7 has been attributed to severity of COVID-19 in young adults (74), in which protection or early administration of MoAbs (REGN-CoV-2) may limit morbidity and mortality. Similarly, Ran Binding Protein 2 (RANBP2) mutation has been associated with fulminant necrotizing encephalitis of influenza (79), in which early prophylactic use of Tamiflu may prevent complication and fatality.

(2) Rapid diagnosis and interrupting viral spread. Rapid diagnostic tests have made early detection and interruption of disease progression and viral transmission possible (148, 149, 152, 153, 155). In an Ebola outbreak, an RDT made a ring vaccination possible that provided 100% vaccine efficacy in the immediate vaccinees (152, 153). Early administration of MoAbs or convalescent plasma has also shown effectiveness on the limitation of disease progression in Ebola (140), SARS-CoV-1 (141), MERS-CoV (142), and SARS-CoV-2 (154, 175–177). It is also postulated that a combination of neutralizing MoAbs and anti-virus agent may induce a synergistic effect (178). The early diagnosis followed by early treatment with MoAbs or convalescent plasma in 72 hours has been shown to reduce viral load, hospitalization and disease progression of COVID-19 (175–177).

(3) Targeting cytokine storm by immunoregulation. Different emerging infections may induce variant types of cytokine storm (87, 92, 97, 120, 158) to which immunotherapies with anti-cytokine and/or immune regulatory therapies have been proposed to rescue the patients with cytokine storm (114, 159–161). It is, however, postulated that aiming at a single target of one cytokine action may be ineffective, and sequential targeting may be required for eliminating the cytokine storm (178). A combined regimen with circulating supports by ECMO and eliminating cytokines by CRRT (156, 157, 162) may be beneficial.

(4) Targeting intracellular signal pathways. Hyperactivation of MAPK pathway and CK2 (casein kinase 2)-phosphorylation have been associated with SARS-CoV-1 and SARS-CoV-2 infections (97, 137), and inhibition of p38 activation or CK2 activation has been shown to decrease viral replication and cytokine induction (137, 174).

(5) Homeostasis of host milieu. The abnormal virus-host interactions for fulminant inflammation on emerging infections may not only depend on viral mutation and host genetic variants, but also host milieu: interior environment, such as imbalances of vitamins and microbiota, and external environment, such as temperature, humidity and protection equipment. For instance, maintenance of host interior homeostasis on vitamins (e.g. vitamin D, retinoids, vitamin K2) and metabolites of microbiota, which provide anti-virus properties and/or better Treg responses for anti-inflammatory reactions (179–184), may regulate immunity and reduce mortality of an emerging infection.

## Author Contributions

BY completed the literature review, performed several studies on immunopathogenesis of enterovirus 71, and drafted the manuscript. KY made the scheme for writing this perspective article and revised the manuscript for final submission. All authors contributed to the article and approved the submitted version.

## Funding

This study was supported in part by a grant MMH109-005 from Mackay Memorial Hospital, Taiwan; and a grant MOST 109-2811-B195-503 from Ministry of Science and Technology, Taiwan.

## Conflict of Interest

The authors declare that the research was conducted in the absence of any commercial or financial relationships that could be construed as a potential conflict of interest.
